# Acceleration of Inner-Pairing Product Operation for Secure Biometric Verification †

**DOI:** 10.3390/s21082859

**Published:** 2021-04-19

**Authors:** Seong-Yun Jeon, Mun-Kyu Lee

**Affiliations:** Department of Electrical and Computer Engineering, Inha University, Incheon 22212, Korea; roland.korea@gmail.com

**Keywords:** inner-pairing product, functional encryption, biometric verification

## Abstract

With the recent advances in mobile technologies, biometric verification is being adopted in many smart devices as a means for authenticating their owners. As biometric data leakage may cause stringent privacy issues, many proposals have been offered to guarantee the security of stored biometric data, i.e., biometric template. One of the most promising solutions is the use of a remote server that stores the template in an encrypted form and performs a biometric comparison on the ciphertext domain, using recently proposed functional encryption (FE) techniques. However, the drawback of this approach is that considerable computation is required for the inner-pairing product operation used for the decryption procedure of the underlying FE, which is performed in the authentication phase. In this paper, we propose an enhanced method to accelerate the inner-pairing product computation and apply it to expedite the decryption operation of FE and for faster remote biometric verification. The following two important observations are the basis for our improvement—one of the two arguments for the decryption operation does not frequently change over authentication sessions, and we only need to evaluate the product of multiple pairings, rather than individual pairings. From the results of our experiments, the proposed method reduces the time required to compute an inner-pairing product by 30.7%, compared to the previous best method. With this improvement, the time required for biometric verification is expected to decrease by up to 10.0%, compared to a naive method.

## 1. Introduction

Biometric recognition is the automated recognition of individuals based on their biological and behavioral characteristics. Biometric recognition has two types [1]: biometric identification and biometric verification. Biometric identification is a process that searches against a biometric enrolment database to find and return the biometric reference identifier(s) attributable to a single user. Conversely, biometric verification is a process that confirms a biometric claim through biometric comparison. In a biometric verification system, a user can make a biometric claim to a biometric characteristic examiner. When a user claims that he or she is the source of a specified biometric reference, the examiner may verify this claim by performing a biometric comparison. With the recent advances in mobile technologies, biometric verification is being adopted in many smart devices as a tool for authenticating their owners. This technique is used not only to unlock devices but also permit users to run security-critical applications, such as financial services [2].

For biometric verification, the biometric data of a user should be stored first during biometric enrolment. The stored biometric data are referred to as a biometric reference, and they are stored using a data structure called a biometric template [1]. However, biometric characteristics are unique and unchangeable; this implies that leakage of these characteristics may cause more critical privacy issues than the compromise of existing passwords and personal identification numbers (PINs). Furthermore, users’ biometric templates are often compromised, especially for mobile devices [3]. Therefore, a biometric verification method that ensures the security of the biometric template must be developed [4,5].

Several proposals are available in the literature to secure biometric templates without additional hardware support such as ARM Trust Zone [6] and Apple Secure Enclave [7]. First, there are methods to convert biometric data using noninvertible transforms, such as cancelable biometrics [8,9] and fuzzy commitment [10]. However, these methods have a problem of decreasing recognition accuracy owing to the conversion process. In addition, there are many cases where one-way transformations are analyzed and inverted successfully [11,12,13].

There are also biometric key generation techniques for biometric template protection [14,15,16]. Using these methods, a unique and high-entropy key can be generated from the user’s biometric input on the fly. These methods have a very desirable property that the user’s biometric template does not need to be stored in the device. However, to achieve both goals of providing a sufficient level of recognition rate and effectively generating biometric keys, these methods require additional tools. For example, the electrocardiogram-based biometric key generation method in [14] requires for helper data to be stored. According to [16], the use of helper data is not desirable. The electroencephalography (EEG)-based method in [15] requires high-bandwidth data with more than 60 channels. It was pointed out in [17] that in most commercially available EEG devices, less than 20 channels are provided. The fingerprint-based biometric key generation method in [16] requires an additional device such as a smart card. On the contrary, if a remote server is available, storing the biometric template on the remote server outside the device is another option for biometric verification without the need for additional tools or performance degradation [18,19,20,21]. In these methods, the server functions as a secure repository. This approach is also suitable when a user wants to be granted access to a particular remote service using an authentication server (e.g., online banking). However, this approach can raise another privacy issue because the remote server can learn the user’s biometric characteristics. In other words, an honest-but-curious server may try to recover the user’s biometric features using the user’s stored template. In addition, attackers who intrude into the server may obtain the biometric features of the legitimate user. These features may be used to impersonate the legitimate user in another system [19,20,21]. Therefore, an encryption scheme that encrypts biometric data and makes the server examine the similarity in encrypted data is necessary for a privacy-preserving biometric verification.

Meanwhile, functional encryption (FE) is an encryption scheme in which possessing a secret key allows one to obtain only the result of f(x) from a ciphertext E(x), but not learn any information about the data *x*, where the secret key is related to the function *f* and the ciphertext E(x) is the encryption of data *x*. Hence, FE is considered a suitable scheme for constructing a privacy-preserving biometric verification system [22,23,24]. As FE requires considerable computation, extensive research has been conducted to make FE more practical, particularly when the evaluated function is an inner product of a plaintext vector **x** with a vector **y** encoded in the function *f* [25,26,27,28,29]. Kim et al. proposed a practical inner product FE scheme with a function-hiding property, which implies that not only **x** but also **y** remain hidden [22]. They also provided a reference code implemented in Python and evaluated the required durations for main operations, such as key generation, encryption, and decryption. According to their measurement, the dominant operations took up to several seconds on a desktop PC, and this indicates that FE may guarantee a practical level of performance. Kim et al.’s scheme is very suitable as a base scheme for a privacy-preserving biometric verification system, but its decryption operation requires an inner-pairing product operation [22], which is its most time-consuming part. An inner-pairing product is the product of multiple pairings, and its inputs are two vectors comprising points on a certain elliptic curve [30]. There are several optimization techniques for the inner-pairing product [31,32,33,34]. Scott suggested to share underlying operations for multiple pairings [31] and the validity of this approach was verified in [32,33]. Costello et al. proposed to apply precomputation to accelerate pairing operations [34].

In this paper, we propose an improved method for accelerating inner-pairing product computation by combining the shared computation techniques and precomputation. The experimental results indicate that the proposed method reduces the time required to compute an inner-pairing product by up to 30.7%. To cope with the situation where the memory is not sufficient to store all the precomputed data, we also propose an adaptive method that can adjust the number of elements to be precomputed and stored. According to our analysis, the performance of the proposed method can be fine-tuned adaptively according to the storage capacity. Furthermore, we demonstrate that the proposed method is suitable for application to a remote biometric verification system using FE, where one of the two inputs to the inner-pairing product operation is not frequently changed. Using the proposed method, we can assume that the performance of biometric verification will be enhanced by 9.0–10.0%.

### 1.1. Related Works

In 2005, Scott proposed three ideas to optimize the inner-pairing product computation for Tate pairing by sharing the common operations among the pairings [31]. In 2006, Granger et al. showed that Scott’s method can also be applied to the inner-pairing product for Ate pairing [32]. In 2015, Zavattoni et al. proposed an optimized method to compute the products of optimal Ate pairings on the BN curve [33]. Meanwhile, in 2010, Costello et al. proposed a precomputation method to accelerate pairing computation when one argument of the pairing is fixed [34]. The more times the pairing is called, the more gain is obtained in the execution time, at the small expense of memory to store the precomputed elements. Recently, the pairing operations are being adopted as a crucial operation for many applications, such as privacy-preserving applications [35,36] and non-interactive zero-knowledge proofs [37,38].

There have been various research results in the literature for secure remote biometric verification [39,40,41,42,43,44,45,46,47,48]. In 2016, Cheon et al. proposed a homomorphic encryption (HE)-based protocol to support encrypted Hamming distance computation required for iris recognition [40]. In the same year, Im et al. [41] proposed an HE-based protocol to support encrypted Euclidean distance computation for palm print authentication [49]. In 2018, Gunasinghe and Bertino proposed a secure face verification protocol based on zero-knowledge proof of knowledge (ZKPK). To perform their protocol, a trusted execution environment (TEE) should be equipped on the device. In the same year, Droandi et al. [48] proposed a multi-party computation-based biometric matching protocol using SPDZ protocol [50]. Although their protocol was very fast, it required a high communication cost for preprocessing. In 2020, Im et al. proposed a secure face verification system that guarantees a real-time authentication performance on a smartphone [2]. They evaluated the performance of their system with two experiments; (1) an experiment involving 30 users in a real-world environment and (2) an experiment using the public face datasets CFP [51] and ORL [52]. In this paper, we consider the data presented in [2] as the criteria to evaluate the performance of the proposed method, since the result in [2] is one of the most up-to-date practical works in the literature of privacy-preserving biometric authentication. A more extensive survey of FE-based privacy-preserving applications can be found in [35].

### 1.2. Contributions

In this paper, we propose a method for accelerating inner-pairing product computation by combining the shared computation techniques and precomputation. To be precise, our contributions are as follows:

- We propose a new algorithm that accelerates inner-pairing product computation when one of the two input vectors are fixed. For each element in this fixed vector, we apply the precomputation method that was originally proposed for a single pairing in [34]. In addition, we reduce the overall computational complexity by sharing overlapping operations for multiple pairings, as in [31].
- To handle the situation where the memory is not sufficient to store all the precomputed data for all vector elements, we also propose an adaptive method that can adjust the number of precomputed elements. Therefore, the performance of the proposed method can be fine-tuned adaptively by selectively storing the precomputed elements according to the storage capacity.
- We demonstrate that the proposed method can significantly accelerate the FE-based biometric verification process. In particular, we exploit the fact that the values related to a biometric template can be precomputed when registered as the template is stored once and repeatedly used without any change.

## 2. Preliminaries

### 2.1. Remote Biometric Verification System

**Figure 1:**
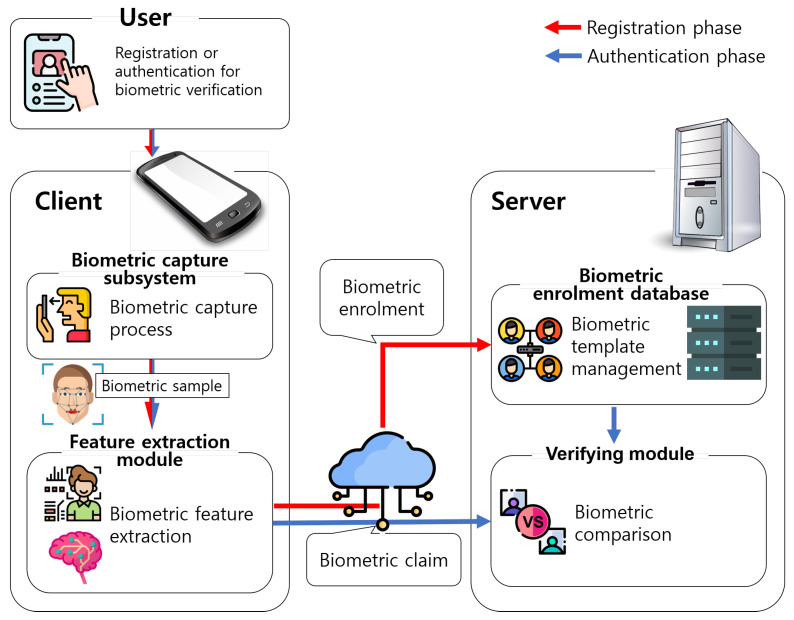
Generic structure of a remote biometric verification system.

In this study, we focus on biometric verification among the two types of biometric recognition. A biometric characteristic examiner can use a remote biometric verification system to authenticate users. Figure 1 shows the generic structure of the remote biometric verification system [1]. For better understanding, we will explain the structure with a facial verification system as an example. The system consists of a client for the user and a remote server for the biometric characteristic examiner. We can consider a smartphone as a typical example of the client. The following are the steps required to register a user’s biometric data.

1. A user presents a biometric characteristic to a biosensor in a biometric capture subsystem, and the subsystem runs its biometric capture process to acquire a biometric sample. For facial verification, the user’s smartphone camera takes a picture of the user’s face. In addition to the biometric capture process, a biometric acquisition process is performed when required. The biometric acquisition process includes segmentation, quality control, and other preprocessing steps. For facial verification, various image processing techniques, such as face detection, alignment and frontalization, can be applied.
2. A client passes the biometric sample to the feature extraction module. The module attempts to extract a biometric feature from the biometric sample. For face verification, deep neural networks are frequently used [53,54].
3. The client performs biometric enrolment. To be precise, the client sends the extracted biometric feature to the server, and the server stores the biometric feature as a biometric reference to a biometric enrolment database. In the database, biometric references are managed using a data structure referred to as a biometric template.

In the authentication phase, the client performs the biometric capture and feature extraction processes, which are the same as the first and second steps of the above biometric registration phase. Then, the client sends the extracted biometric feature as the biometric claim to be used for verification. The server delivers the biometric feature from the client to its verifying module. This claimed feature is referred to as a biometric probe. The module loads the biometric reference (i.e., the stored biometric template) of the claimant for biometric comparison. Biometric comparison is the similarity estimation between the biometric reference and the biometric probe. For example, if the biometric feature is represented as a vector, the similarity can be estimated by computing either the Hamming or Euclidean distance between the two feature vectors. Based on the result of biometric comparison (i.e., the comparison score) and a decision policy including a threshold, whether the biometric probe and biometric reference have the same biometric source is determined. If this is affirmative, the server accepts the claimant as an authenticated user.

However, as explained in the introduction, the above approach can be a threat to user’s privacy, because the server may try to recover the user’s biometric features from the stored template. Section 2.4 will briefly explain the countermeasure against this threat using functional encryption.

### 2.2. Barreto—Naehrig Curve (BN Curve)

If a non-supersingular elliptic curve over $F_{p}$ contains a subgroup whose embedding degree *k* is not substantially large, it is called a pairing-friendly curve. In other words, computations in the field $F_{p^{k}}$ are feasible. Barreto and Naehrig presented a method to construct pairing-friendly elliptic curves of prime order and embedding degree k=12 [55], whose curve form is $E\left(F_{p}\right):y^{2}=x^{3}+b$, with b≠0. For non-zero *t*, they parameterized the order *n* of the elliptic curve group and the characteristic *p* as follows:

(1) $$n=36t^{4}+36t^{3}+18t^{2}+6t+1$$

(2) $$p=36t^{4}+36t^{3}+24t^{2}+6t+1$$

The order of a point P∈E is the least non-zero integer *r* such that [r]P=∞, where [r]P is the sum of *r* terms equal to *P* [56]. Therefore, the fact that *n* is a prime implicitly indicates that *r* is equal to *n*, and *r* is also prime.

### 2.3. Pairing

#### 2.3.1. Cryptographic Pairing

Pairing is defined as a map $e:G_{1}\times G_{2}\to G_{T}$ for additive groups $G_{1}$, $G_{2}$ and a multiplicative group $G_{T}$ [57]. The orders of $G_{1}$, $G_{2}$, and $G_{T}$ are the prime number *r*. The identity elements of these groups are denoted by $0_{G_{1}}$, $0_{G_{2}}$, and $1_{G_{T}}$, respectively. Furthermore, the pairing should have the following two properties:

- Bilinearity For all $g_{1}\in G_{1}$, $g_{2}\in G_{2}$, and $a,b\in Z_{r}$,
  (3) $$e\left(\left[a\right]g_{1},\left[b\right]g_{2}\right)=e{\left(g_{1},g_{2}\right)}^{ab}.$$
- Non-degeneracy For $g_{1}\neq 0_{G_{1}}$, $g_{2}\neq 0_{G_{2}}$
  (4) $$e\left(g_{1},g_{2}\right)\neq 1_{G_{T}}.$$

In addition to the above two mathematical properties, cryptographic pairing requires the following property [57]:

- Computability The map *e* can be efficiently computed.

The most efficient cryptographic pairings are constructed using an elliptic curve *E*, which is defined over a finite field $F_{q}$. Specifically, $G_{1}$ and $G_{2}$ are subgroups of the rational points of an elliptic curve *E* defined over an extension $F_{q^{k}}$ of $F_{q}$. Furthermore, $G_{T}$ is the group $\left(F_{q^{k}}^{*},\times \right)$, where the group law is given by the field multiplication on $F_{q^{k}}$. We define the tuple $\left(r,G_{1},G_{2},G_{T},g_{1},g_{2},e\right)$ as a *bilinear environment* of cryptographic pairing.

An inner-pairing product $e_{prod}$ is defined by the following equation for two vectors P = $(P_{1},$ $P_{2},$ …, $P_{d})$ $\in G_{1}^{d}$ and Q = $(Q_{1},$ $Q_{2},$ …, $Q_{d})$ $\in G_{2}^{d}$:

(5) $$e_{prod}\left(P,Q\right)=\prod_{j=1}^{d}e\left(P_{j},Q_{j}\right)$$

#### 2.3.2. Miller’s Algorithm and Final Exponentiation

The Weil pairing was first introduced by André Weil in 1940 [58]. It plays an important role in the theoretical study of the arithmetic of elliptic curves and Abelian varieties [58]. Miller presented an algorithm in 2004 that efficiently computes Weil pairing, which is the first practical pairing computation method [59]. Since then, most pairings, including Weil Pairing, have been designed based on Miller’s algorithm for efficient operation. The basic Miller’s algorithm takes a pair of elements of the elliptic curve subgroups $G_{1}$ and $G_{2}$, both of whose orders are the prime order *r*, and repeats a series of processes as much as the bit length *m* of *r*. This loop is referred to a *Miller loop*. For any point *U*, *V* on the elliptic curve and the element $X\in G_{1}$, $Y\in G_{2}$, line equation $L_{U,V}\left(X,Y\right)$ is defined as the equation of the line passing through *U* and *V*, whereas tangent equation $T_{U}\left(X,Y\right)$ is defined as the tangent to the point *U*. Miller’s algorithm includes multiplication and squaring operations on $G_{T}$, addition and multiplication operations on $G_{1}$ or $G_{2}$, and evaluation of the line and tangent equations. Miller’s algorithm is used to compute not only Weil pairing but also many other cryptographic pairings, such as the Tate pairing or Tate variant pairings [60,61]. Thus, a special operation referred to as *final exponentiation* is performed to force the result of the Miller loop to be a unique value for the multiplicative group $G_{T}$ [57]. In other words, final exponentiation must be performed after the Miller loop to obtain the correct operation result.

#### 2.3.3. Optimal Ate Pairing on the BN Curve

The Ate pairing [62,63] and its variations [64,65,66] are simply optimized versions of the Tate pairing using Frobenius endomorphism [67]. In 2008, Vercauteren proposed optimal pairings and optimized the Miller loop of Ate pairing, which uses Frobenius endomorphism on a pairing-friendly elliptic curve [67]. In 2010, Beuchat et al. presented an implementation for the optimal Ate pairing of [67] on the BN curve [68]. They reported that the performance of Ate pairing is optimized by setting *t* of the BN curve as $2^{62}-2^{54}+2^{44}$. Algorithm 1 represents the algorithm for calculating the optimal Ate pairing [68], where π(Q) is the Frobenius map of *Q* and $\pi^{2}\left(Q\right)=\left(\pi \cdot \pi \right)\left(Q\right)$.
**Algorithm 1** Optimal Ate pairing on the BN curve.**Input:**  s=6t+2, m= the bit length of *s*, $P\in G_{1}$, $Q\in G_{2}$ **Output:**  e(P,Q)
1:Write *s* in signed binary form, $s=\sum_{i=0}^{m-1}s[i]2^{i}$ with s[i]∈{−1,0,1}2:T←Q,f←13:**for**i←m−2**down to** 0 **do**4:    $f\leftarrow f^{2}\cdot L_{T,T}\left(P\right),T\leftarrow 2T$5:    **if** s[i]=1 **then**6:        $f\leftarrow f\cdot L_{T,Q}\left(P\right),T\leftarrow T+Q$7:    **else if** s[i]=−1 **then**8:        $f\leftarrow f\cdot L_{T,-Q}\left(P\right),T\leftarrow T-Q$9:    **end if**10:**end for**11:$R\leftarrow \pi \left(Q\right),f\leftarrow f\cdot L_{T,R}\left(P\right),T\leftarrow T+R$12:$R\leftarrow \pi^{2}\left(Q\right),f\leftarrow f\cdot L_{T,-R}\left(P\right),T\leftarrow T-R$13:$f\leftarrow f^{(p^{12}-1)/r}$14:**return***f*


### 2.4. Function-Hiding Inner Product Encryption

Inner product encryption (IPE) is an FE whose function *f* is the inner product of the input vector x with the vector y encoded in the function *f*. That is, IPE performs f(x)=〈x,y〉, by using a secret key associated with vector y and the ciphertext associated with vector x as inputs.

Meanwhile, if the FE guarantees that the data associated with its function *f* remain hidden as well as x, we confirm that an FE has a function-hiding property. For example, the associated data may be vector y for IPE, and a privacy-preserving biometric verification system using IPE should be equipped with the function-hiding property as the biometric data should be securely handled both in the registration and authentication phases [69].

Hereinafter, we use $\prod_{IPE}$, a function-hiding IPE with practical performance proposed by Kim et al. [22]. For λ∈N, d∈N, and the range of the inner product *S*, the function-hiding IPE is defined as $\prod_{IPE}$ = (IPE.Setup, IPE.KeyGen, IPE.Encrypt, IPE.Decrypt), where each operation is defined as follows:

- $IPE.Setup(1^{\lambda },S)$
  1. Select a bilinear environment $\left(r,G_{1},G_{2},G_{T},g_{1},g_{2},e\right)$ according to the security parameter λ.
  2. Choose a matrix $B\leftarrow {\mathrm{GL}}_{d}\left(Z_{r}\right)$, where ${\mathrm{GL}}_{d}\left(Z_{r}\right)$ refers to a group of d×d square matrix, where each element belongs to the finite field $Z_{r}$ and an inverse matrix exists.
  3. Compute $B^{⋆}\leftarrow \det\left(B\right)\cdot {\left(B^{-1}\right)}^{⊤}$.
  4. Output the public parameter $pp=(G_{1},G_{2},G_{T},r,e,S)$ and the master secret key $msk=(pp,g_{1},g_{2},B,B^{⋆})$.
- IPE.KeyGen(msk,y)
  1. Choose a uniformly random element $\alpha \overset{R}{\leftarrow }Z_{r}$.
  2. Using the master key msk and the vector $y\in Z_{r}^{d}$, output the secret key $sk=\left(K_{1},K_{2}\right)=\left(\left[\alpha \cdot \det\left(B\right)\right]g_{1},\left[\alpha \cdot y\cdot B\right]g_{1}\right)$, s.t. $K_{2}\in G_{1}^{d}$.
- IPE.Encrypt(msk,x)
  1. Choose a uniformly random element $\beta \overset{R}{\leftarrow }Z_{r}$
  2. Using the master key msk and the vector $x\in Z_{r}^{d}$, output the ciphertext $ct=\left(C_{1},C_{2}\right)=\left(\left[\beta \right]g_{2},\left[\beta \cdot x\cdot B^{⋆}\right]g_{2}\right)$, s.t. $C_{2}\in G_{2}^{d}$.
- IPE.Decrypt(pp,sk,ct)
  1. Using the public parameter pp, the secret $sk=(K_{1},K_{2})$, and the ciphertext $ct=(C_{1},C_{2})$, compute $D_{1}=e\left(K_{1},C_{1}\right)$ and $D_{2}=e_{prod}\left(K_{2},C_{2}\right)$.
  2. Find a solution *z* for $D_{1}^{z}=D_{2}$ If this *z* exists, it satisfies z=〈x,y〉. Output *z* if it exists; otherwise, output ⊥, indicating that a solution does not exist.

In this study, we use $\prod_{IPE}$ to construct a privacy-preserving biometric verification system. The two vectors **x** and **y** are encoded to ensure that they represent the biometric probe and biometric reference, respectively. Furthermore, the authors of [22] suggested methods to encode a biometric feature vector to either **x** or **y**; thus, the Hamming and the Euclidean distance between two biometric feature vectors can be calculated using the inner product 〈x,y〉. Using this method, we can encrypt all biometric data transmitted to the server as well as the stored biometric template. The biometric comparison is performed on the encrypted biometric data. However, the comparison score is output as a plain value to ensure that the verification module can decide regarding the authenticity of the claimant. In summary, a biometric verification system that keeps all biometric data from leaking the biometric characteristics even during the biometric comparison can be constructed using $\prod_{IPE}$.

## 3. Existing Optimization Techniques for Computing Pairing

The inner-pairing product can be performed in a naive manner to calculate $e(P_{j},Q_{j})$ for all *j* and multiply them all. We call this approach the *Naive method*. However, this native method can be improved based on two directions of research. We briefly review them in this section.

### 3.1. Optimal Ate Pairing Product on the BN Curve

In 2005, Scott proposed three ideas to optimize the Naive method for Tate pairing [31].

1. In the case of a modular multiplicative inverse operation, a simultaneous inversion operation [70] can be applied.
2. During the computation of Miller’s algorithm, the squaring operation on $G_{T}$ (e.g., in line 4 of Algorithm 1) can be shared.
3. The final exponentiation operation can be shared.

In 2006, Robert Granger et al. reported that the performance of inner-pairing product computation for Ate pairings can be improved by applying the second and third ideas [32]. In 2015, Eric Zavattoni et al. implemented an optimized method to compute the products of optimal Ate pairings on the BN curve, which we call the *Product method* in this paper [33].

Algorithm 2 demonstrates how the Product method is computed in the bilinear environment of Section 2.3.3.

### 3.2. Fixed Argument Pairings

In 2010, Costello et al. proposed a method to compute a pairing using precomputation when one argument of the pairing is fixed [34]. Algorithm 3 demonstrates the application of precomputation to *Q* for the optimal Ate pairing, adopting the method in [34] when $Q\in G_{2}$ is fixed. Algorithm 4 presents the pairing computation procedure when *P* and $Q^{\prime }$ are given as inputs, where $Q^{\prime }$ is a precomputed tuple based on *Q*. The main idea of this precomputation-based method is that the line and the tangent equation in the Miller loop of the optimal Ate pairing can be precomputed with only *Q* without *P* to obtain the gradient λ and constant *c* of the equations. π can also be computed in advance with only *Q*. Thus, it is also included in precomputation.

During online computation of the pairing, the precomputed $Q^{\prime }$, rather than *Q*, is applied to the linear equation, tangent equation, and π. The *Fixed-Q method* refers to an inner-pairing product method that replaces the individual pairing of the Naive method to the online computation of Fixed-Q pairing.
**Algorithm 2** Products of optimal Ate pairings on the BN curve (Product method).**Input:** s=6t+2, m= the bit length of *s*, $P_{j}\in G_{1}$, $Q_{j}\in G_{2}$, where *j* is 1,…,d **Output:** $e_{prod}\left(P,Q\right)$
1:Write *s* in signed binary form, $s=\sum_{i=0}^{m-1}s[i]2^{i}$ with s[i]∈{−1,0,1}2:f←13:**for**j←1**to***n***do**4:    $T_{j}\leftarrow Q_{j}$5:**end for**6:**for**i←m−2**down to** 0 **do**7:    $f\leftarrow f^{2}$8:    **for** j←1 **to** *d* **do**9:        $f\leftarrow f\cdot L_{T_{j},T_{j}}\left(P_{j}\right),T_{j}\leftarrow \left[2\right]T_{j}$10:        **if** s[i]=1 **then**11:           $f\leftarrow f\cdot L_{T_{j},Q_{j}}\left(P_{j}\right),T_{j}\leftarrow T_{j}+Q_{j}$12:        **else if** s[i]=−1 **then**13:           $f\leftarrow f\cdot L_{T_{j},-Q_{j}}\left(P_{j}\right),T_{j}\leftarrow T_{j}-Q_{j}$14:        **end if**15:    **end for**16:**end for**17:**for**j←1**to***d***do**18:    $R\leftarrow \pi \left(Q_{j}\right),f\leftarrow f\cdot L_{T_{j},R}\left(P_{j}\right),T_{j}\leftarrow T_{j}+R$19:    $R\leftarrow \pi^{2}\left(Q_{j}\right),f\leftarrow f\cdot L_{T_{j},-R}\left(P_{j}\right),T_{j}\leftarrow T_{j}-R$20:**end for**21:$f\leftarrow f^{(p^{12}-1)/r}$22:**return***f*
**Algorithm 3** Fixed-Q precomputation.**Input:** s=6t+2, m= the bit length of *s*, $Q\in G_{2}$ **Output:** $Q^{\prime }=\left(G_{DBL},G_{ADD},\pi_{Q},{\pi^{2}}_{Q}\right)$
1:Write *s* in signed binary form, $s=\sum_{i=0}^{m-1}s[i]2^{i}$ with s[i]∈{−1,0,1}2:$T\leftarrow Q,G_{DBL}\leftarrow \left\{\emptyset \right\},G_{ADD}\leftarrow \left\{\emptyset \right\}$3:**for**i←m−2**down to** 0 **do**4:    Compute λandc, such that $y_{Q}+\lambda x_{Q}+c$ is the line tangent to *T*5:    T←[2]T6:    Append (λ,c) to $G_{DBL}$7:    **if** s[i]=1 **then**8:         Compute λandc, such that $y_{Q}+\lambda x_{Q}+c$ is the line joining *T* and *Q*9:         T←T+Q10:        Append (λ,c) to $G_{ADD}$11:    **else if** s[i]=−1 **then**12:        Compute λandc, such that $y_{-Q}+\lambda x_{-Q}+c$ is the line joining *T* and −Q13:        T←T−Q14:        Append (λ,c) to $G_{ADD}$15:    **end if**16:**end for**17:R←π(Q)18:Compute λandc, such that $y_{R}+\lambda x_{R}+c$ is the line joining *T* and *R*19:$\pi_{Q}\leftarrow \left(\lambda ,c\right)$20:$R\leftarrow -\pi^{2}\left(Q\right)$21:Compute λandc, such that $y_{R}+\lambda x_{R}+c$ is the line joining *T* and *R*22:${\pi^{2}}_{Q}\leftarrow \left(\lambda ,c\right)$23:$Q^{\prime }\leftarrow \left(G_{DBL},G_{ADD},\pi_{Q},{\pi^{2}}_{Q}\right)$24:**return**$Q^{\prime }$


**Algorithm 4** Fixed-Q pairing.**Input:** s=6t+2, m= the bit length of $s,P\in G_{1}$, $Q^{\prime }=\left(G_{DBL},G_{ADD},\pi_{Q},{\pi^{2}}_{Q}\right)$ (the precomputation tuple for *Q*, where $Q\in G_{2}$) **Output:** e(P,Q)1:Write *s* in signed binary form, $s=\sum_{i=0}^{m-1}s[i]2^{i}$ with s[i]∈{−1,0,1}2:f←1,cnt←13:**for**i←m−2**down to** 0 **do**4:    $\lambda ,c\leftarrow G_{DBL}\left[i\right]$5:    Compute $g\leftarrow (y_{P}+\lambda x_{P}+c)$6:    $f\leftarrow f^{2}\cdot g$7:    **if** s[i]≠0 **then**8:         $\lambda ,c\leftarrow G_{ADD}\left[cnt\right]$9:         Compute $g\leftarrow (y_{P}+\lambda x_{P}+c)$10:        cnt←cnt+111:        f←f·g12:    **end if**13:**end for**14:$\lambda ,c\leftarrow \pi_{Q}$15:Compute $g\leftarrow (y_{P}+\lambda x_{P}+c)$16:f←f·g17:$\lambda ,c\leftarrow {\pi^{2}}_{Q}$18:Compute $g\leftarrow (y_{P}+\lambda x_{P}+c)$19:f←f·g20:$f\leftarrow f^{(p^{12}-1)/r}$21:**return***f*


## 4. Proposed Method

In this section, we present our proposed method to efficiently compute an inner-pairing product. The proposed method combines two previous approaches and adopts both precomputation and shared computation techniques. We call our method the *Fixed-Q Product* method. This method is a revised version of the method presented in the preliminary version of this paper [71] and the Master’s Thesis of the first author [72].

Algorithm 5 presents the detailed procedure of the Fixed-Q Product method. In the input of the Fixed-Q Product method, $Q^{\prime }$ is used instead of Q, unlike the Product method. In other words, all elements of Q in Algorithm 2 are now used to obtain $Q^{\prime }$ using the Fixed-Q precomputation (Algorithm 3) in advance.

We initialize a few variables in lines 1–5 of Algorithm 5 prior to performing the Miller loop. The parameter *s* is expanded as a signed binary form. The variable *f* for accumulating the products of the pairings is set to 1, and all elements of the array cnt for $G_{ADD}$ are initialized to 1.

After initialization, the Miller loop runs in lines 6–20 of Algorithm 5. Unlike in a single pairing, the Miller loop has the form of a nested loop as it computes multiple pairings. In the case of the Naive method, the inside loop should have been executed many times, depending on the number of inputs *d*. The nested loop structure of Algorithm 5 is the same as that of the Product method to share the squaring operation. In other words, the code of lines 7–19 is repeated by the length of *s* by using *i*. In each iteration of this outer loop, lines 9–18 are repeated based on the number of inputs *d* using *j* in the inside loop. Through application of this nested loop, the proposed method can improve the performance of the Fixed-Q method similar to how the Product method improves the Naive method. Furthermore, we can describe the effect of our method in the aspect of the amount of online computation. In other words, unlike the Product method, the proposed method performs the Fixed-Q pairing using $Q_{j}^{\prime }$ in the code of lines 9–18. To support this improvement, each array element cnt[j] plays the role of the variable cnt of Algorithm 4.
**Algorithm 5** Fixed-Q Product method.**Input:** s=6t+2, m= the bit length of *s*, $P=\{\left(P_{1},\ldots ,P_{d}\right)|P_{j}\in G_{1}\}$, $Q^{\prime }=\{\left(Q_{1}^{\prime },\ldots ,Q_{d}^{\prime }\right)|Q_{j}^{\prime }$ is the precomputation tuple for $Q_{j}\in G_{2}\}$ **Output:** $e_{prod}\left(P,Q\right)$1:Write *s* in signed binary form, $s=\sum_{i=0}^{m-1}s[i]2^{i}$ with s[i]∈{−1,0,1}2:f←13:**for**j←1**to***d***do**4:    cnt[j]←15:**end for**6:**for**i←m−2**down to** 0 **do**7:    $f\leftarrow f^{2}$8:    **for** j←1 **to** *d* **do**9:        $G_{DBL},G_{ADD}\leftarrow Q_{j}^{\prime }$10:        $\lambda ,c\leftarrow G_{DBL}\left[i\right]$11:        Compute $g\leftarrow (y_{P}+\lambda x_{P}+c)$12:        f←f·g13:        **if** s[i]≠0 **then**14:           $\lambda ,c\leftarrow G_{ADD}\left[cnt\left[j\right]\right]$15:           Compute $g\leftarrow (y_{P}+\lambda x_{P}+c)$16:           cnt[j]←cnt[j]+117:           f←f·g18:        **end if**19:    **end for**20:**end for**21:**for**j←1**to***d***do**22:    ${\pi^{\prime }}_{Q},{\left(\pi^{\prime }\right)}_{Q}^{2}\leftarrow Q_{j}^{\prime }$23:    $\lambda ,c\leftarrow {\pi^{\prime }}_{Q}$24:    Compute $g\leftarrow (y_{P}+\lambda x_{P}+c)$25:    f←f·g26:    $\lambda ,c\leftarrow {{\left(\pi^{\prime }\right)}^{2}}_{Q}$27:    Compute $g\leftarrow (y_{P}+\lambda x_{P}+c)$28:    f←f·g29:**end for**30:$f\leftarrow f^{(p^{12}-1)/r}$31:**return***f*


The Frobenius map and final exponentiation should be applied to the optimal Ate pairing after the Miller loop. As the operation of the Frobenius map is only related to *Q*, all the Frobenius maps of individual pairings can be included in the Fixed-Q precomputation. Therefore, we apply the precomputed Frobenius maps to the proposed method by using $\pi_{Q}^{\prime }$ and ${\left(\pi^{\prime }\right)}_{Q}^{2}$ from $Q_{j}^{\prime }$ in lines 21–29. As mentioned in Section 3.1, the final exponentiation is a shareable operation. Thus, the final exponentiation can be performed only once after the Miller loop (in line 30).

It should be noted that the speedup of the proposed method is obtained at the expense of additional memory to store $Q^{\prime }$. The exact amount of memory required to store the *d* tuples $Q_{1}^{\prime },\ldots ,Q_{d}^{\prime }$ of $Q^{\prime }$ will be analyzed in the next section. To handle the situation where the memory budget is very tight, we propose an adaptive method that adjusts the number of precomputed tuples according to the storage capacity. Algorithm 6 is this modified version for the situation where only the memory for k(≤d) precomputed tuples is available. Without loss of generality, we assume that only the precomputed tuples $Q_{1}^{\prime },\ldots ,Q_{k}^{\prime }$ are given. Therefore, Algorithm 6 takes as input these tuples as well as $Q_{k+1},\ldots ,Q_{d}$, the field elements for the non-precomputed portion. Algorithm 6 can be viewed as the combination of Algorithm 5 and Algorithm 2. Its main loop is the same as that of Algorithm 5. However, it has lines 6–8, lines 23–30, and lines 41–44, i.e., the routines to handle the operations corresponding to non-precomputed elements. By adjusting the parameter *k*, the algorithm can be adapted to the current memory capacity. That is, Algorithm 6 has a time-memory trade-off. The relation between the number of precomputed tuples and the speed will be precisely analyzed in the next section.
**Algorithm 6** Adaptive method.**Input:** s=6t+2, m= the bit length of *s*, $P=\{\left(P_{1},\ldots ,P_{d}\right)|P_{j}\in G_{1}\}$, $Q^{\prime \prime }=\{\left(Q_{1}^{\prime },\ldots ,Q_{k}^{\prime },Q_{k+1},\ldots ,Q_{d}\right)$$|Q_{j}^{\prime }$ is the precomputation tuple for $Q_{j}\in G_{2},1<k\leq d\}$ **Output:** $e_{prod}\left(P,Q\right)$1:Write *s* in signed binary form, $s=\sum_{i=0}^{m-1}s[i]2^{i}$ with s[i]∈{−1,0,1}2:f←13:**for**j←1**to***k***do**4:    cnt[j]←15:**end for**6:**for**j←k+1**to***d***do**7:    $T_{j}\leftarrow Q_{j}$8:**end for**9:**for**i←m−2**down to** 0 **do**10:    $f\leftarrow f^{2}$11:    **for** j←1 **to** *k* **do**12:        $G_{DBL},G_{ADD}\leftarrow Q_{j}^{\prime }$13:        $\lambda ,c\leftarrow G_{DBL}\left[i\right]$14:        Compute $g\leftarrow (y_{P}+\lambda x_{P}+c)$15:        f←f·g16:        **if** s[i]≠0 **then**17:           $\lambda ,c\leftarrow G_{ADD}\left[cnt\left[j\right]\right]$18:           Compute $g\leftarrow (y_{P}+\lambda x_{P}+c)$19:           cnt[j]←cnt[j]+120:           f←f·g21:        **end if**22:    **end for**23:    **for** j←k+1 **to** *d* **do**24:        $f\leftarrow f\cdot L_{T_{j},T_{j}}\left(P_{j}\right),T_{j}\leftarrow \left[2\right]T_{j}$25:        **if** s[i]=1 **then**26:           $f\leftarrow f\cdot L_{T_{j},Q_{j}}\left(P_{j}\right),T_{j}\leftarrow T_{j}+Q_{j}$27:        **else if** s[i]=−1 **then**28:           $f\leftarrow f\cdot L_{T_{j},-Q_{j}}\left(P_{j}\right),T_{j}\leftarrow T_{j}-Q_{j}$29:        **end if**30:    **end for**31:**end for**32:**for**j←1**to***k***do**33:    ${\pi^{\prime }}_{Q},{\left(\pi^{\prime }\right)}_{Q}^{2}\leftarrow Q_{j}^{\prime }$34:    $\lambda ,c\leftarrow {\pi^{\prime }}_{Q}$35:    Compute $g\leftarrow (y_{P}+\lambda x_{P}+c)$36:    f←f·g37:    $\lambda ,c\leftarrow {{\left(\pi^{\prime }\right)}^{2}}_{Q}$38:    Compute $g\leftarrow (y_{P}+\lambda x_{P}+c)$39:    f←f·g40:**end for**41:**for**j←k+1**to***d***do**42:    $R\leftarrow \pi \left(Q_{j}\right),f\leftarrow f\cdot L_{T_{j},R}\left(P_{j}\right),T_{j}\leftarrow T_{j}+R$43:    $R\leftarrow \pi^{2}\left(Q_{j}\right),f\leftarrow f\cdot L_{T_{j},-R}\left(P_{j}\right),T_{j}\leftarrow T_{j}-R$44:**end for**45:$f\leftarrow f^{(p^{12}-1)/r}$46:**return***f*


## 5. Performance Analysis

### 5.1. Theoretical Analysis

In this subsection, we analyze the expected amount of computation and storage required for the Naive, Product, Fixed-Q, and the proposed Fixed-Q Product methods. First, we denote the total amount of computation required for the basic optimal Ate pairing algorithm (Algorithm 1) and the Fixed-Q pairing algorithm (Algorithm 4) as $C_{basic}$ and $C_{fixed}$, respectively. As explained in Section 3.2, $C_{fixed}$ is significantly smaller than $C_{basic}$. We also denote the amount of computation required for a squaring operation on $G_{T}$ and the final exponentiation as $C_{sqr}$ and $C_{fin}$, respectively. Subsequently, the amount of computation required to compute an inner-pairing product (5) of two *d*-dimensional vectors can be expressed as follows:

- Naive method
  (6) $$C_{basic}\times d$$
- Product method
  (7) $$\left(C_{basic}-C_{sqr}-C_{fin}\right)\times d+C_{sqr}+C_{fin}$$
- Fixed-Q method
  (8) $$C_{fixed}\times d$$
- Fixed-Q Product method (proposed)
  (9) $$\left(C_{fixed}-C_{sqr}-C_{fin}\right)\times d+C_{sqr}+C_{fin}$$

Noticeably, the computational costs of all methods are represented as linear functions in *d*. However, comparing (6) and (7), we can observe that the term $C_{sqr}+C_{fin}$ was moved from the slope to the constant intercept part, thus, significantly reducing the slope. The same relation holds between (8) and (9). In particular, the cost reduction of the proposed method over the Fixed-Q method (and that of the Product method over the Naive method) is $\left(d-1\right)\left(C_{sqr}+C_{fin}\right)$. Consequently, the proposed method is expected to be the fastest among the four methods when d≥2.

The amount of computation for the Fixed-Q method and the proposed method is reduced at the expense of memory to store the precomputed elements. In other words, a time-memory trade-off occurs. To estimate the additional memory required for storing the precomputed elements, we expressed the bit length of the data structure $Q^{\prime }$ that represents the precomputation table. $Q^{\prime }$ is a tuple composed of $\left(G_{DBL},G_{ADD},\pi_{Q},{\pi^{2}}_{Q}\right)$. According to Algorithm 3, $G_{DBL}$ and $G_{ADD}$ are the arrays with their elements in $F_{p}\times F_{p}$. Whenever the code of line 6 in Algorithm 3 is run, the number of elements in the $G_{DBL}$ array is, thus, increased by one, and this line is repeated by m−1 times. Thereafter, $G_{DBL}$ will contain m−1 elements in $F_{p}\times F_{p}$. The codes of line 10 and 14 also increase the length of $G_{ADD}$ by one. However, these lines are executed only when the corresponding element s[i] in the signed binary representation of *s* is non-zero. For given $t=2^{62}-2^{52}+2^{44}$, the number of non-zero terms in the signed binary representation of *s* is exactly seven. Therefore, $G_{ADD}$ will finally contain seven elements in $F_{p}\times F_{p}$. Finally, the codes of lines 19 and 22 are executed once for $\pi_{Q}$ and ${\pi^{2}}_{Q}$. Therefore, $\pi_{Q},{\pi^{2}}_{Q}\in F_{p}\times F_{p}$. As *m*, the bit length of s=6t+2, is 65, we can observe that $Q^{\prime }\in {\left(F_{p}\times F_{p}\right)}^{(m-1)+7+2}={\left(F_{p}\times F_{p}\right)}^{73}$, and the bit length of $Q^{\prime }$ can be expressed as 73×2×l=146l, where *l* denotes the bit length of prime *p*. In other words, 146l bits are additionally required to store the precomputed data $Q^{\prime }$ in Algorithm 3 and use it in Algorithm 4. For Algorithm 5, we need *d* tables $Q_{1}^{\prime },\ldots ,Q_{d}^{\prime }$, requiring 146dl bits of the precomputation storage.

### 5.2. Experimental Results

To verify the performance improvement, we implemented the proposed method as well as the three previous methods, and then we compared their performance in terms of execution time and memory usage. This experiment was conducted on a desktop PC with an Intel(R) Core(TM) i7-7700 CPU @ 3.60GHz, 16GB memory, and Ubuntu Desktop 16.04 LTS. The program was written in C++. In particular, GMP 6.1.2 [73], MCL 1.05 [74], and NTL 11.3.2 [75] libraries were applied for algebraic operations.

According to the theoretical analysis in Section 5.1, the amount of computation for each method can be expressed as a linear equation in the number *d* of the input pairs. The slope of the linear equation and the value of the *y*-intercept can be estimated by measuring the execution times of the component operations. Therefore, we measured the durations required for a squaring operation and final exponentiation and as those for pairing operations. Table 1 presents the result of this measurement. The figures in Table 1 are the average of the execution times in 10,000 executions with a random input for each operation, and the unit of execution time is $10^{6}$ CPU clocks (Mclk).

Using the measured data, we estimated the slopes and *y*-intercepts in the linear Equations (6)–(9) in the previous subsection. The cost of the squaring operation $C_{sqr}$ can be estimated as m×(the execution time for a single squaring operation), where *m* (i.e., the bit length of s=6t+2) is calculated as 65 given that $t=2^{62}-2^{54}+2^{44}$ for the optimal Ate pairing. Consequently, we obtained the following expressions for the execution times of the four inner-pairing product methods, which are also summarized in Table 2:

- Naive method:
  (10) $$C_{Naive}=1.595d$$
- Product method:
  (11) $$C_{Prod}=\left(1.595-0.008\times 65-0.629\right)d+0.008\times 65+0.629=0.464d+1.131$$
- Fixed-Q method:
  (12) $$C_{FixedQ}=1.411d$$
- Fixed-Q Product method (proposed):
  (13) $$C_{FixedQProd}=\left(1.411-0.008\times 65-0.629\right)d+0.008\times 65+0.629=0.280d+1.131$$

**Figure 2:**
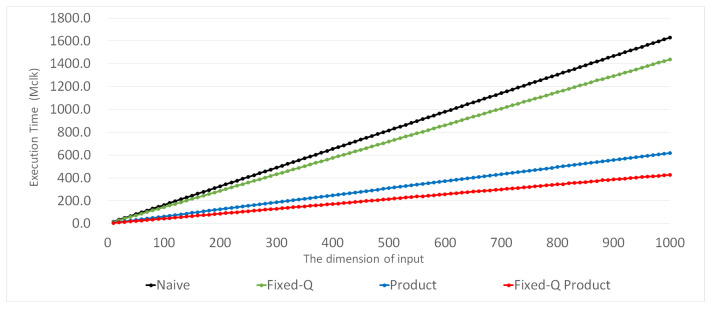
Performance comparison according to the dimension of input for each method.

**Figure 3:**
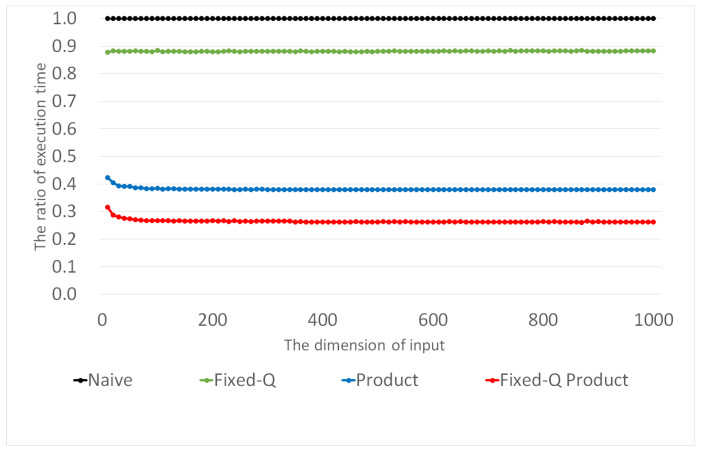
Comparison of the performance ratio of other methods to compute inner-pairing products compared to the Naive method.

We also verified the validity of the expressions given above by directly measuring the execution times of the inner-pairing product computation. Furthermore, we measured these execution times, increasing *d* from 10 to 1000 by 10. For each combination, we measured the execution time of each method 1000 times. Figure 2 presents the average execution times of the four inner product methods. Certainly, the execution times of the four methods increase almost linearly with *d*. Figure 3 presents the relative ratio of the execution time of each method to that of the Naive method. We can observe that the ratio remains as a constant for each method, except the region for small *d*, where the influences of constant terms in (7) and (9) are non-negligible. Please note that the amounts of computation in the Naive and Fixed-Q methods (Equations (6) and (8)) are proportional to *d*, but those in the Product and Fixed-Q Product methods are not exactly proportional to *d*. However, when *d* gets sufficiently large, the constant terms in (7) and (9) almost do not affect the overall performance, and (7) and (9) become almost proportional to *d*. For this region with sufficiently large *d*, the relative ratio of the execution time is essentially the ratio of the slopes. For example, the ratio of the Fixed-Q Product method over the Naive method is approximately $\left(C_{fixed}-C_{sqr}-C_{fin}\right)/\left(C_{basic}\right)$ for large *d*, whereas it is originally $\left(\left(C_{fixed}-C_{sqr}-C_{fin}\right)d+\left(C_{sqr}+C_{fin}\right)\right)/\left(C_{basic}d\right)$. This explains the slightly bent portions in the curves for the Product and Fixed-Q Product methods in Figure 3.

In other words, four horizontal lines are almost parallel to the *d*-axis. The constant ratios are 0.881, 0.381, and 0.264 for the Fixed-Q, Product, and proposed methods, respectively. This implies that the proposed method improves the performance of the Naive, Fixed-Q, and Product methods by 3.8, 3.3, and 1.4 times, respectively. If we compare the proposed method with the best previous method, Product, we see that the proposed method reduces the execution time of the Product method by 1−0.264/0.381≈30.7%.

**Figure 4:**
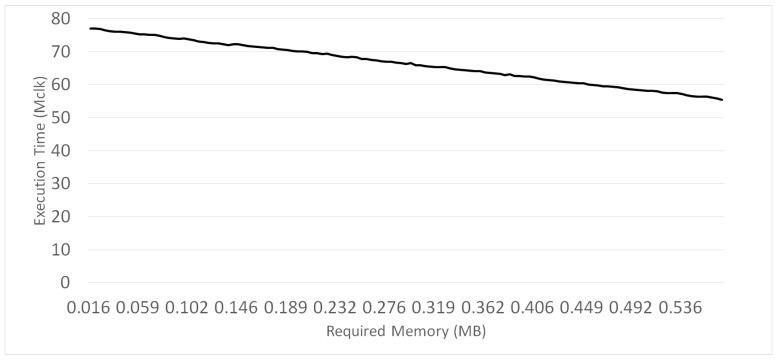
Time-memory trade-off of the adaptive method.

The amount of storage space required for each method was also measured and analyzed. The software module used in this study uses a 256-bit data type to express an element in $F_{p}$. Each element in $G_{1}$ is a point on an elliptic curve defined over $F_{p}$. Therefore, it is composed of two elements in $F_{p}$ to represent x and y coordinates. Each element in $G_{2}$ is a point on an elliptic curve defined over an extension field. Therefore, it is composed of four elements in $F_{p}$. Subsequently, the sizes of the data structures to handle the elements in $G_{1}$ and $G_{2}$ are 512(=2×256) and 1024(=4×256) bits, respectively. The data structure for a precomputation table $Q^{\prime }$ consumes 146×256=37,376 bits. When an inner-pairing product is computed with two *d*-dimensional vectors using the proposed method, 37,376d bits are required for the precomputation table. For example, considering that d=130, which is a typical value for our biometric verification application, this amount corresponds to only 0.6 MB. As explained in Section 4, only a subset of precomputed elements may be computed if the storage is not sufficient. Figure 4 demonstrates the time-memory trade-off of Algorithm 6 for various values of *k* when d=130. The x-axis represents the amount of available memory. *k* varies from 0 to 130. When k=130, approximately 0.6 MB is required. As the graph shows, the execution time of Algorithm 6 linearly decreases according to the increase in the amount of memory.

Finally, we verify whether the Fixed-Q and Fixed-Q Product methods consume additional communication bandwidth for the transmission of the precomputation tables. When the inner-pairing product operation is applied to privacy-preserving remote biometric verification, this operation is performed by the receiver (i.e., by the remote authentication server) to conduct IPE.Decrypt with two input vectors P = $(P_{1},$$P_{2},$…,$P_{d})$$\in G_{1}^{d}$ and Q = $(Q_{1},$$Q_{2},$…,$Q_{d})$$\in G_{2}^{d}$. The second vector, Q, is related to the stored biometric template. Therefore, it is transmitted in the registration phase, and the required precomputation can be conducted on the server side. In other words, Algorithm 3 is performed for each element in Q by the server. Therefore, additional communication bandwidth is not required.

## 6. Application

As explained in Section 2.4, if FE is applied to a remote biometric verification system, the client may encrypt and securely transmit the user’s biometric data, the server may store the encrypted biometric template, and the biometric comparison may be performed while all biometric data remain encrypted. In this section, we construct a simple facial verification system using $\prod_{IPE}$ and describe how the FE can be applied to a remote biometric verification system, following the idea presented in [22]. We also demonstrate that the proposed method significantly improves the facial verification performance. For biometric comparison, the server uses Euclidean distance as the metric for the similarity between two feature vectors, which is the most widely used approach in biometric authentication. Furthermore, a vector encoding method proposed by Kim [22] is adopted to perform this comparison in the ciphertext domain. When two *d*-dimensional feature vectors x and y are given as a biometric reference and biometric probe, respectively, the similarity score is defined as ${∥x-y∥}^{2}$ (i.e., the square of the Euclidean distance between x and y). To compute ${∥x-y∥}^{2}$ using $\prod_{IPE}$, the following three operations are defined:

- EncodeX(msk,x)
  1. Construct a (d+2)-dimensional vector $x^{\prime }={(∥x∥}^{2},-2x_{1},-2x_{2},\ldots ,-2x_{d},1)$ from $x=(x_{1},\ldots ,x_{d})$.
  2. Output $ct=IPE.Encrypt(msk,x^{\prime })$.
- EncodeY(msk,y)
  1. Construct a (d+2)-dimensional vector $y^{\prime }=(1,y_{1},y_{2},\ldots ,y_{d}{,∥y∥}^{2})$ from $y=(y_{1},\ldots ,y_{d})$.
  2. Output $sk=IPE.KeyGen(msk,y^{\prime })$.
- Euclid(pp,sk,ct)
  1. Calculate z=IPE.Decrypt(pp,sk,ct).
  2. Output *z*. (*z* satisfies $z=\langle x^{\prime },y^{\prime }\rangle ={(∥x∥}^{2}-2x_{1}y_{1}-2x_{2}y_{2}-\ldots -2x_{d}y_{d}+{∥y∥}^{2}{)=(∥x∥}^{2}-2\langle x,y\rangle +{∥y∥}^{2}{)=∥x-y∥}^{2}).$

In our biometric verification system, IPE.Encrypt (i.e., EncodeX) will be used to protect the biometric template in the registration phase. Meanwhile, IPE.KeyGen (i.e., EncodeY) will be used to protect the biometric probe of the claimant in the authentication phase. We might have used the IPE functions in other ways (i.e., IPE.KeyGen for registration and IPE.Encrypt for authentication). However, we made the above choice owing to the following reason: the stored biometric template does not change frequently after registration, whereas the biometric probe for authentication changes every session. Thus, applying precomputation to the stored template is suitable. According to Algorithm 5, the precomputation is only applicable to the second argument of $e_{prod}\left(P,Q\right)$, that is, Q, whose elements are from $G_{2}$. According to the description of $\prod_{IPE}$ in Section 2.4, this Q should be $C_{2}$, which is computed by IPE.Encrypt.

**Figure 5:**
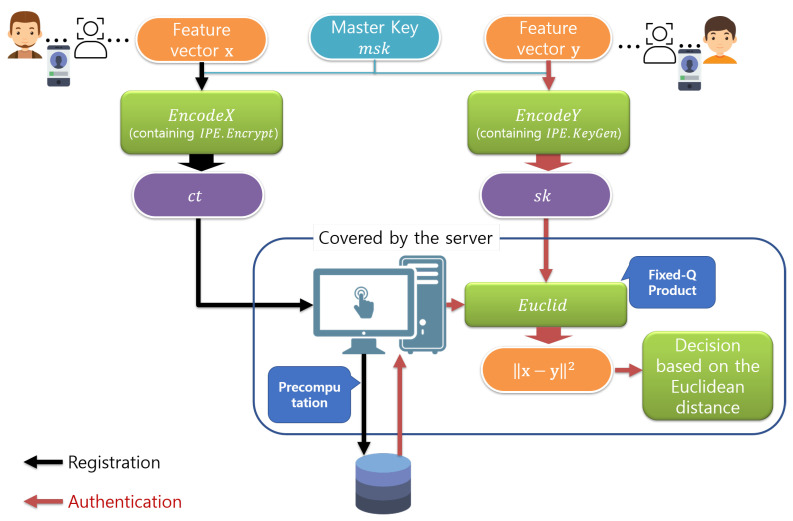
Simple facial verification system that uses $\prod_{IPE}$ with the proposed method.

Figure 5 shows the simple facial verification system that uses the above three operations. EncodeX and EncodeY are performed by the client. Meanwhile, Euclid is performed by the server and includes an inner-pairing product for IPE.Decrypt. In other words, if the inner-pairing product can be accelerated, we can also improve the performance of Euclid.

As shown in Figure 5, the client transmits a user’s encrypted feature vector as a biometric reference to the server, and then the server stores the encrypted vector as a biometric template in the registration phase. Using the proposed method, the server can perform the Fixed-Q precomputation on all the elements of the biometric template and use the precomputed data to accelerate the inner-pairing product computation in Euclid.

To estimate the effect of the proposed method in the performance of a remote biometric verification system, we emulated a remote facial verification system where the client comprises an image processing module that processes the face image and produces a feature vector, and a cryptographic module for the EncodeX and EncodeY operations. The server performs the biometric comparison using a cryptographic module implementing the Euclid operation. We did not actually implement the image processing module, but emulated the one provided in [2]. We brought a part of the experimental results for image processing time in [2], and actually measured the durations for $\prod_{IPE}$ operations. Combining both data, we estimated the overall time for biometric verification. To measure the times for $\prod_{IPE}$ operations, we used the same mobile device and PC as used in [2]. Table 3 presents our experimental results. The face image processing column covers the entire image processing procedure, i.e., from biometric capture and acquisition process and feature extraction. In [2], four datasets were provided—Auto, Guide, CFP, and ORL. Each dataset uses 128-dimensional feature vectors in common. Therefore, we measured the performance of EncodeY with 128-dimensional feature vectors. However, we did not compare the performance of EncodeX as it is called only once for registration. In particular, we measured the performance of Euclid by dividing it into two parts, Pairing and DLP. Pairing is the part that consists of a single pairing operation for $D_{1}$ and $e_{prod}$ for $D_{2}$ in IPE.Decrypt. For comparison, we measured the performance of the inner-pairing product, $e_{prod}$, using the proposed and Naive methods. DLP is the part that finds a solution to the discrete logarithm problem $D_{1}^{z}=D_{2}$. The total execution time for authentication comprises face image processing, EncodeY, and Euclid, and presents the data when the Naive method and the proposed method are applied. The last column provides the ratio of reduced time over the Naive method (i.e., $(1-\frac{Total\;(proposed)}{Total\;(Naive)})\times 100$%). From Table 3, the proposed Fixed-Q Product method reduces the time for Euclid operation by $\left(1-\frac{15.66+120.30}{58.86+120.30}\right)\times 100$% = 24.1% compared to the Naive method, and the overall authentication time is also expected to be reduced by 9.0–10.0%. Regarding the required memory, note that approximately 0.6 MB of precomputation memory should be available to fully exploit Algorithm 5 when a 128-dimensional feature vector is used, i.e., d=130. If multiple users are registered to the authentication server, the amount of required memory will be proportional to the number of registered users. For example, 600 MB is required to store the precomputation data for 1000 users. However, if we use Algorithm 6, we may permit more users, slightly degrading the authentication speed.

### Security Analysis

To show that our FE-based facial verification system manages and processes the biometric information in a secure and privacy-compliant manner, we evaluate our system based on the requirements of biometric information protection, i.e., irreversibility, unlinkability, renewability, and performance [76,77].

- Irreversibility: the irreversibility of our system depends on the security of $\prod_{IPE}$. Please note that the template is not stored in the client device. Therefore, the only concern is the possibility of template recovery on the server side. However, the $\prod_{IPE}$ guarantees that the server cannot learn any information about the stored ciphertext, except its inner product with another ciphertext. Therefore, the encrypted biometric information is protected by $\prod_{IPE}$.
- Unlinkability: Our procedure for template encryption, i.e., IPE.Encrypt, involves a uniformly random component β. Consequently, it may produce completely different ciphertexts even when the same biometric information is encrypted. Therefore, it is not possible to link two or more biometric templates encrypted using $\prod_{IPE}$.
- Renewability: Every call to the template encryption procedure IPE.Encrypt generates a completely different ciphertext even for the same user using a random parameter β. Therefore, it can create multiple, independently transformed biometric templates.
- Performance: According to the ISO/IEC 19795-1 standard [77], we consider the biometric accuracy as a criterion of performance. It is straightforward that the accuracy of the proposed system is exactly the same as that of the underlying biometric verification system, because the proposed system does not revise the feature extraction process. It only encrypts the extracted features. Therefore, the biometric similarity score computed from ct=EncodeX(msk,x) and sk=EncodeY(msk,y) is exactly the same as that computed from the original x and y.

Finally, we remark that the biometric information is still protected even when the precomputation on the biometric template is applied to the system. According to Algorithm 3, precomputation is performed only using s,m, and *Q*, where *s* and *m* are publicly known parameters. *Q* ($Q_{j}$ in later algorithms) is an element in the encrypted biometric template vector. Therefore, when the server performs Algorithm 3 for precomputation, it does not obtain any additional information about the feature value encoded in the template. Consequently, whether the server performs the precomputation or not does not affect the security of the system. The same holds for the case where the server is compromised by an attacker.

## 7. Discussion

In this paper, we proposed a method to accelerate the inner-pairing product operation for secure biometric verification. The proposed Fixed-Q Product method is a method that optimizes inner-pairing product computation by applying precomputation. We also applied the new inner-pairing product method to design a secure biometric verification system. To verify the feasibility of the proposed method, we emulated a simple facial verification system comprising a client and a server. Our analysis results indicate that the new inner-pairing product method accelerates biometric authentication. However, the proposed method has a time-memory trade-off. The reduction in the amount of computation for the proposed method is obtained at the expense of memory to store the precomputed elements. Although the new method requires more memory than the previous methods, the amount of the additional memory is not considerable. Moreover, there are no changes in the bandwidth requirement for communication as the precomputation is performed on the server side. Furthermore, we also provide the adaptive method where the amount of precomputed elements is parameterized. Therefore, a server can choose to apply the proposed method by itself while a client is not aware of it. In other words, selectively tuning the performance of the system is possible.

We remark that noise may affect the security and performance of a biometric authentication system [78]. Therefore, we consider the effect of noise on the proposed method. First, note that the proposed system does not revise the feature extraction procedure, but it only encrypts the extracted features. Therefore, the noise-robustness of the underlying system is maintained even after applying our method, if only the integrity of a transmitted or stored ciphertext is guaranteed. On the contrary, if a ciphertext is modified, the server will be able to notice this change immediately, because the modification of even a single bit will make the data invalid. The probability that a modified element becomes a valid field element constituting a ciphertext is almost zero. In summary, the proposed method does not affect the noise-robustness of a biometric authentication system.

Finally, the proposed method is suitable not only for remote biometric verification systems but also for any FE-based privacy-preserving applications where the evaluated function is an inner product and one of the two inputs of the inner product is entered offline [35,36]. Furthermore, the proposed method can be applied not only to FE-based systems but also to the other systems involving inner-pairing products, such as non-interactive zero-knowledge proofs [37,38].

## Figures and Tables

**Table 1:** Execution times of the operations constituting an inner-pairing product.

Operation Name	Execution Time (Mclk)
Squaring on $G_{T}$	0.008
Final exponentiation	0.629
Basic optimal Ate pairing (Algorithm 1)	1.595
Fixed-Q pairing (Algorithm 4)	1.411

**Table 2:** Execution times of the inner-pairing product computation for various methods.

Methods	Expected Cost	Measured Cost
Naive	$C_{basic}\times d$	1.595d
Product	$\left(C_{basic}-C_{sqr}-C_{fin}\right)\times d+C_{sqr}+C_{fin}$	0.464d+1.131
Fixed-Q	$C_{fixed}\times d$	1.411d
**Proposed**	$\left(C_{fixed}-C_{sqr}-C_{fin}\right)\times d+C_{sqr}+C_{fin}$	0.280d+1.131

**Table 3:** Performance improvement in the facial verification system using the proposed method compared to the Naive method (times in ms).

Biometric Dataset	Face Image Processing [2]	EncodeY	Euclid			Total (Naive)	Total (Proposed)	Ratio
			Pairing (Naive)	Pairing (Proposed)	DLP			
Auto	193.27	106.47	58.86	15.66	120.30	478.90	435.70	9.0 %
Guide	157.47					443.10	399.90	9.7%
CFP	150.15					435.78	392.58	9.9%
ORL	147.33					432.96	389.76	10.0%

## Abbreviations

The following abbreviations are used in this manuscript:

FE	Functional encryption
PIN	Personal identification number
EEG	Electroencephalography
HE	Homomorphic encryption
ZKPK	Zero-knowledge proof of knowledge
TEE	Trusted execution environment
BN curve	Barreto-Naehrig curve
IPE	Inner product encryption
